# A new and efficient methodology for olefin epoxidation catalyzed by supported cobalt nanoparticles

**DOI:** 10.3762/bjoc.17.46

**Published:** 2021-02-22

**Authors:** Lucía Rossi-Fernández, Viviana Dorn, Gabriel Radivoy

**Affiliations:** 1 INQUISUR-CONICET, Departamento de Química, Universidad Nacional del Sur, Avenida Alem 1253, Bahía Blanca, B8000CPB, Argentina

**Keywords:** alkenes, cobalt nanoparticles, epoxides, oxidation, TBHP

## Abstract

A new heterogeneous catalytic system consisting of cobalt nanoparticles (CoNPs) supported on MgO and *tert*-butyl hydroperoxide (TBHP) as oxidant is presented. This CoNPs@MgO/*t*-BuOOH catalytic combination allowed the epoxidation of a variety of olefins with good to excellent yield and high selectivity. The catalyst preparation is simple and straightforward from commercially available starting materials and it could be recovered and reused maintaining its unaltered high activity.

## Introduction

Olefin oxidation reactions are key synthetic transformations in the production of oxygenated chemicals of high interest for both academic and industrial applications [1]. Among them, allylic oxidation and olefin epoxidation constitute fundamental tools for the synthesis of homoallylic alcohols or α,β-unsaturated carbonyl compounds, and epoxides, respectively. In particular, epoxides are pivotal building blocks for the synthetic chemists because they are present in many important (bio)organic compounds and also allow access to more functionalized or complex structures through different chemical transformations on the reactive oxirane ring [2–12].

Despite many methodologies for the synthesis of epoxides have been reported, efficient and selective epoxidation of olefins remains a challenge. Due to safety and environmental issues, traditional methods involving the use of stoichiometric amounts of harmful oxidants (for example, peroxosulfates [13] or organic peracids [14]) have been replaced by the use of greener oxidizing agents as molecular oxygen, hydrogen peroxide or *tert*-butyl hydroperoxide (TBHP) [14–17]. However, using any of these oxidants alone results in considerable low reactivity and selectivity in olefin epoxidation reactions. Thus, several transition-metal-based catalytic methods have been developed, most of them using expensive or scarce metals (Au or Pd [18–21], groups IV and V metal oxides [22–23]) and mainly through homogeneous catalytic processes [24–26]. From a practical, economic and environmental point of view, reusable heterogeneous catalysts based on earth-abundant transition metals are much more attractive, especially for industrial applications [27–28].

In recent years, many efforts have been made in finding new catalytic systems based on the use of low cost and abundant non-noble metals, and much attention have been paid to the development of Fe, Mn and mainly Co-based catalysts for olefin epoxidation. Besides their low cost and low toxicity, the choice of these metals is related to their known ability to activate dioxygen in natural processes catalyzed by metal-containing enzymes [29–30]. Despite that various homogeneous [31–32] and heterogeneous [33–37] Co-based catalysts have been applied to the epoxidation of alkenes, however, most of the reported methods lead to unsatisfactory yield, low selectivity, or limited substrate scope. Suib et al. [38] reported on the synthesis of mesoporous Co_3_O_4_ for the catalytic epoxidation of a variety of alkenes as an interesting heterogeneous system. This cobalt oxide mesoporous nanomaterial showed good activity and selectivity to the epoxide product and could be recovered and reused, but the multistep (not straightforward) synthesis of the catalyst and the use of DMF as the solvent (at 100 °C) are main drawbacks of this methodology. DMF has been the solvent of choice in most cobalt-based epoxidation systems and it has been proposed that this solvent could serve as oxygen-transfer agent but, at the same time, could lead to considerable amounts of formamide byproducts [39–40].

On the other hand, the use of supported cobalt nanoparticles as efficient catalysts for the epoxidation of olefins has received increasing attention in the last years. In most cases, a crucial influence of the support (TiO_2_, HAP, CNTs, SBA, SiO_2_), the oxidant agent (molecular oxygen or TBHP) and the solvent (DMF, MeCN, ethyl acetate, DMSO, solvent free) on the activity and selectivity of the nanocatalysts has been noted [27,41–44].

Furthermore, all the reported methodologies use either molecular oxygen together with an aldehyde as a co-reductant, or only a “green” peroxide (H_2_O_2_, TBHP) as the oxidant agent. Among the reported methods that make use of peroxides as oxidants, cobalt nanoparticles supported on CNTs together with TBHP as oxidant for the epoxidation of styrene, gave good selectivity to styrene oxide but conversions were lower than 40% [41]. More recently, Hutchings et al. reported on a Co_3_O_4_/TiO_2_-catalyzed solvent-free version that allowed the use of molecular oxygen together with sub-stoichiometric amounts of TBHP as radical initiator at 80 °C, with conversions lower than 40% and ca. 20% selectivity in the epoxidation of 1-decene as the only substrate tested [42]. On the other hand, among the peroxide-free oxidation processes, a Co_3_O_4_/SBA-16 catalyst for the epoxidation of limonene in AcOEt [43] and a Co/HAP catalyst in DCM as the solvent [44] have been very recently reported. Both of these catalytic systems gave good conversions to the desired epoxides and allowed the use of molecular oxygen as oxidant but require the use of high amounts (400–500% excess) of isobutyraldehyde as co-reductant.

Therefore, there remains a need to develop and improve catalytic systems for alkene epoxidation by using low cost and easy to prepare supported cobalt nanoparticles as reusable heterogeneous catalysts [27] that are wide in substrate scope, active enough and highly selective. As part of our continuing interest in the development of new synthetic methodologies based on the use of catalysis by non-noble transition metal nanoparticles (MNPs) for their application in a wide range of relevant organic transformations [45–52], we report herein our study on the performance of CoNPs/MgO nanocatalyst for olefin epoxidation reactions. Compared to previous reports in the same field, it should be highlighted that our CoNPs/MgO catalyst is readily prepared from low-cost commercially available starting materials, works in acetonitrile as the solvent (thus avoiding the use of toxic DMF), and can be recovered and reused maintaining its high activity.

## Results and Discussion

The supported cobalt nanoparticles tested as catalysts were prepared by fast reduction of anhydrous cobalt(II) chloride with an excess of lithium sand and a catalytic amount of 4,4’-di-*tert*-butylbiphenyl (DTBB, 10 mol %) as electron carrier, in THF as the solvent. Once the reaction mixture turned to black, indicating the formation of the CoNPs, the corresponding support was added and the resulting suspension was stirred for 2 h (see Experimental for details). The CoNPs-based catalysts were ready for use after filtration and drying in an oven at 150 °C for 1 h.

Styrene (**1a**) was chosen as model substrate for testing the activity and selectivity of a variety of CoNPs-based catalysts (Table 1). The catalytic tests were carried out in a sealed glass tube, under a variety of reaction conditions. We started our study by working in DMF as the solvent, under O_2_ (1 atm, balloon) and in the absence of any other oxidant additive. Under these conditions, none of the tested catalysts gave satisfactory results (Table 1, entries 1–4), only the CoNPs/MgO catalyst gave a modest 28% conversion to the desired epoxide **2a** (Table 1, entry 2) together with undesired formamide byproducts, probably coming from DMF decomposition under the reaction conditions. Then, we decided to use acetontrile (MeCN) as the solvent with the CuNPs supported on MgO as catalyst, but a similar result to that obtained in DMF was observed (Table 1, entry 5). The use of H_2_O_2_ as co-oxidant improved the conversion of the starting styrene (**1a**) only for the CoNPs/MgO catalyst, but lead to low selectivity due to the formation of benzaldehyde and benzylic alcohol byproducts (Table 1, entries 6–9). In view of these results, we decided to continue the optimization of the reaction conditions by testing other oxidizing agents and solvents but only for the CoNPs/MgO catalyst. Thus, we found that using this catalyst, in refluxing MeCN as the solvent and TBHP as oxidant (1 equiv in relation to **1a**), resulted in a much better epoxidation system, leading to a 64% conversion with a highly improved selectivity towards the styrene oxide product **2a** (Table 1, entry 10). Under the same reaction conditions, other solvents such as water or dichloromethane gave poorer conversions and selectivities (Table 1, entries 11 and 12).

**Table 1:** Optimization of reaction conditions for the epoxidation of styrene (**1a**).^a^

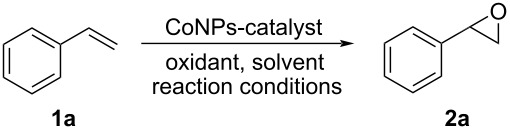					

Entry	Catalyst	Solvent	Oxidant	Conversion^b^ (%)	Selectivity^c^ (%)

1	CoNPs/ZnO	DMF	O_2_	8	30
2	CoNPs/MgO	DMF	O_2_	28	40
3	CoNPs/Celite	DMF	O_2_	0	–
4	CoNPs/CeO_2_	DMF	O_2_	0	–
5	CoNPs/MgO	MeCN	O_2_	22	35
6	CoNPs/ZnO	MeCN	H_2_O_2_	10	50
7	CoNPs/MgO	MeCN	H_2_O_2_	35	45
8	CoNPs/Celite	MeCN	H_2_O_2_	0	–
9	CoNPs/CeO_2_	MeCN	H_2_O_2_	5	ND
10	CoNPs/MgO	MeCN	TBHP	64	94
11	CoNPs/MgO	DCM	TBHP	11	43
12	CoNPs/MgO	H_2_O	TBHP	15	72
**13**	**CoNPs/MgO**	**MeCN**	TBHP	60^d^/**95**^e^/73^f^	**94**

^a^Styrene (**1a**, 0.5 mmol), 50 mg of catalyst in 5 mL of solvent at reflux temperature, 12 h; ^b^GLC yield based on the starting styrene (anisole as internal standard); ^c^selectivity expressed as yield of styrene oxide based on the starting styrene; ^d^reaction performed using 5 mg of catalyst, 93% selectivity; ^e^reaction performed using 10 mg of catalyst, 94% selectivity; ^f^reaction performed using 20 mg of catalyst, 91% selectivity.

Next, we worked on the optimization of the catalyst loading. Thus, when the amount of catalyst was decreased from 50 mg to 20 mg, a higher conversion was observed along with a slight drop in the selectivity towards the epoxide product **2a** (Table 1, entry 13, footnote f). In view of this observation, we then tested the epoxidation reaction by lowering the catalyst loading. As shown in Table 1 (entry 13, footnote e), the optimum amount of catalyst was found to be 10 mg, which corresponds to a cobalt loading of 1.07 mol % in relation to the starting styrene (**1a**), leading to a 95% conversion with excellent selectivity to the styrene oxide product (**2a**).

Additional experiments confirmed the need for the presence of both the CoNPs/MgO catalyst and TBHP as oxidant for the selective epoxidation of styrene (**1a**). Thus, when the reaction was performed without the addition of TBHP, only a 14% conversion to styrene oxide (**2a**) was observed, whereas in the absence of the CoNPs/MgO catalyst the reaction provided a 36% conversion into a 1:1 mixture of styrene oxide (**2a**) and benzaldehyde.

The scope of the method was then analyzed by studying the epoxidation of a variety of alkenes under the optimized conditions. As shown in Table 2, the CoNPs/MgO catalyst proved to be very efficient in the epoxidation of different terminal and internal alkenes, both alkyl- or aryl-substituted ones. Unfortunately, electron-poor olefins, conjugated with C=O groups, gave very low conversion (pulegone, 22%) or did not react under the optimized conditions, even after 24 h of reaction time (methyl cinnamate, methyl acrylate, methyl methacrylate, isobutyl acrylate).

**Table 2:** Epoxidation of various alkenes under the optimized conditions.^a^

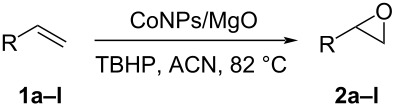				

Entry	Starting alkene	Product	Selectivity (%)^b^	Yield (%)^c^

1	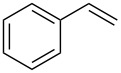 **1a**	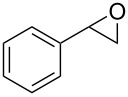 **2a**	94	95
2	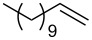 **1b**	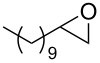 **2b**	100	71
3	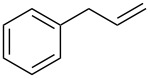 **1c**	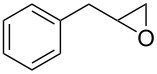 **2c**	88	74^d^
4	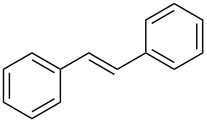 **1d**	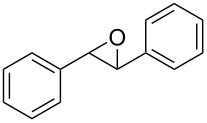 **2d**	100	97
5	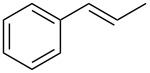 **1e**	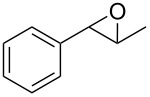 **2e**	95	95
6	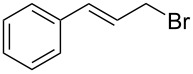 **1f**	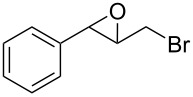 **2f**	74	72^d^
7	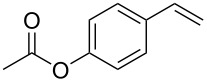 **1g**	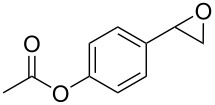 **2g**	78	67^d^
8	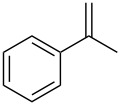 **1h**	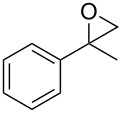 **2h**	73	99
9	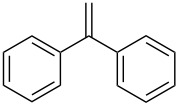 **1i**	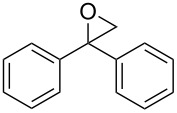 **2i**	92	76
10	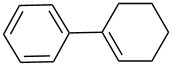 **1j**	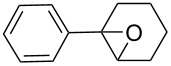 **2j**	81^e^	75
11	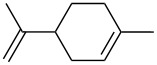 **1k**	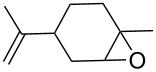 **2k**	70	57^f^
12	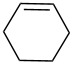 **1l**	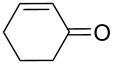 **2l**	86	90

^a^Alkene (0.5 mmol), CoNPs/MgO catalyst (10 mg, 1.07 mol % Co), TBHP (0.5 mmol), in MeCN (5 mL) at 82 °C for 12 h, unless otherwise stated; ^b^selectivity expressed as yield of alkene oxide based on the starting alkene determined by GC–MS; ^c^GLC yield based on the starting styrene (anisole as internal standard); ^d^18 h of reaction time; ^e^together with the corresponding ketone in position 3 (α-oxidation product, 19%); ^f^together with the corresponding diepoxide as byproduct (10%).

For cycloalkenes (Table 2, entries 10–12) different oxidation patterns were observed depending on the alkene structure and/or degree of substitution at the C=C double bond. Thus, 1-phenylcyclohexene (**1j**, Table 2, entry 10), gave the epoxide **2j** as the main product along with 19% of the corresponding allylic ketone in position 3 of the cyclohexenyl moiety (α-oxidation product). On the other hand, the epoxidation of the diene (±)-limonene (**1k**, Table 2, entry 11) took place mainly on the endocyclic C=C double bond with good selectivity, although with a relatively modest conversion. On the contrary, for cyclohexene (**1l**, Table 2, entry 12) the main oxidation product was found to be the corresponding allylic ketone **2l** coming from the α-oxidation of the starting alkene. It is known that the product selectivity in the oxidation of cyclohexene could be influenced by the catalyst and reaction conditions (solvent, temperature, catalyst loading, oxidant) [14].

Based on our experimental observations and those previously made by other authors [15–17,41–44], we assumed that a radical oxidation process could be taking place. To test this assumption, the epoxidation of styrene was carried out under the optimized conditions by adding hydroquinone (5 mg, 0.045 mmol) as radical scavenger. After 8 h of reaction time only 9% conversion into styrene oxide (**2a**) was observed, thus evidencing the presence of radical species as reaction intermediates.

The reusability of the CoNPs/MgO catalyst was then studied for styrene (**1a**) epoxidation as model reaction. After each cycle, the catalyst was separated from the reaction medium by centrifugation and washed several times with acetonitrile. Thus, the catalyst could be recovered and reused in three consecutive cycles, showing no loss of activity, but a drop in selectivity after the first cycle (selectivity/conversion for each cycle: 94/99.5; 78/99; 77/99). The origin of the observed loss in selectivity is difficult to ascertain at this stage, but we think that it could be related to the strong adsorption of some of the reaction products on the catalyst surface. In fact, FTIR analysis of the spent catalyst, after washing it three times with the reaction solvent, showed a weak band at near 1610 cm^−1^ which could be assigned to the presence of benzaldehyde surface species [53] that could be affecting to some extent the selectivity of the catalyst.

The heterogeneous nature of the catalytic process was confirmed by a hot filtration test. For this purpose, under the optimized conditions the epoxidation of styrene (**1a**) was stopped at 20% conversion (20 min), the catalyst filtered off and the resulting filtrate was allowed to react for 6 additional hours. Analysis by GC–MS of the crude reaction mixture revealed a conversion of only 33% into styrene oxide (**2a**), thus confirming the heterogeneous nature of the catalytic method.

**Figure 1:**
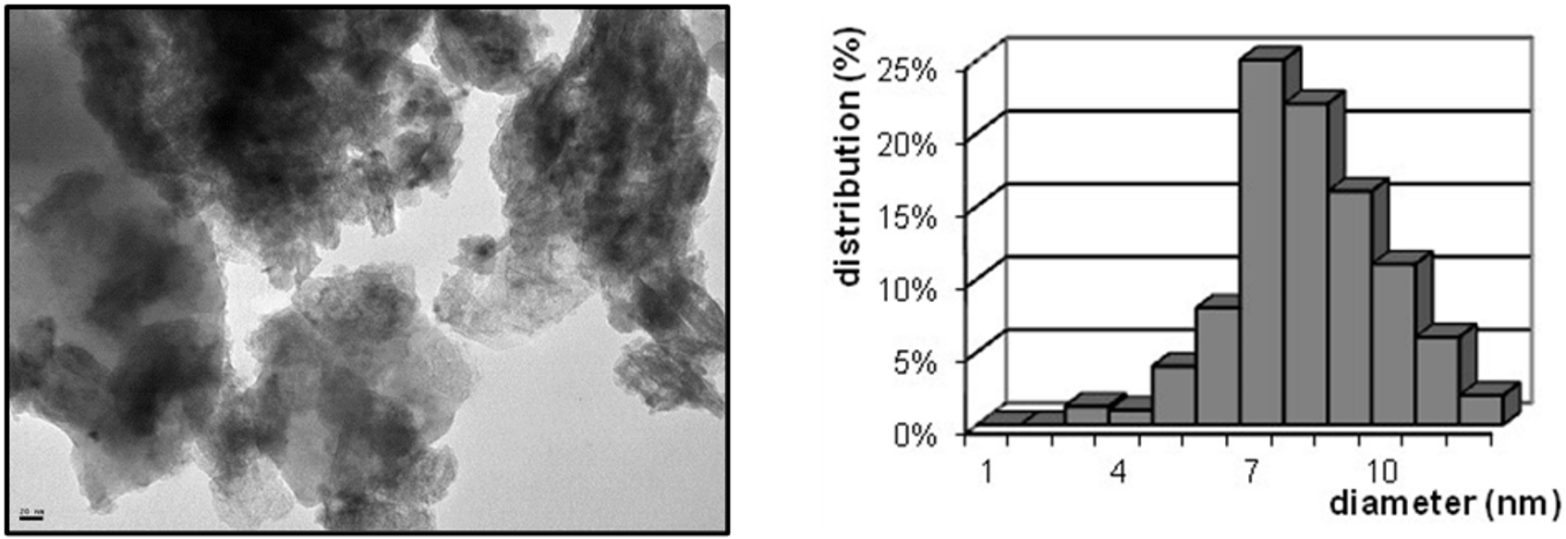
TEM micrograph and size distribution graphic for CoNPs@MgO catalyst (scale bar = 20 nm).

The freshly prepared CoNPs/MgO catalyst was characterized by means of transmission electron microscopy (TEM), energy dispersive X-ray spectroscopy (EDX), inductively coupled plasma atomic emission spectroscopy (ICP-AES), X-ray diffraction (XRD) and X-ray photoelectron spectroscopy (XPS) (see Supporting Information File 1). TEM analysis showed the presence of highly dispersed spherical cobalt nanoparticles, most of them ranging between 6 and 11 nm in size (Figure 1).

EDX analysis in various regions of the sample (Figure S2, Supporting Information File 1) confirmed the presence of cobalt with energy bands of 0.8 (L line), 6.9 and 7.7 keV (K lines). The XRD diffractogram showed only the support diffraction pattern, no diffraction peaks owing to cobalt species could be observed. The cobalt loading fixed to the MgO support was 1.9 wt % as determined by ICP-AES. The analysis of the XPS spectra in the Co 2p region was consistent with the presence of Co^2+^ and Co^3+^, with main binding energy peaks at 779.6 and 780.0 eV, along with satellite signal at approximately 786 eV, which in principle could be ascribed to Co_3_O_4_ species in the catalyst (Figure S4, Supporting Information File 1). Nevertheless, it must be pointed out that the oxidation state of cobalt is difficult to assign from the XPS results, due to the binding energy overlap of the different cobalt oxides [54].

**Scheme 1:**
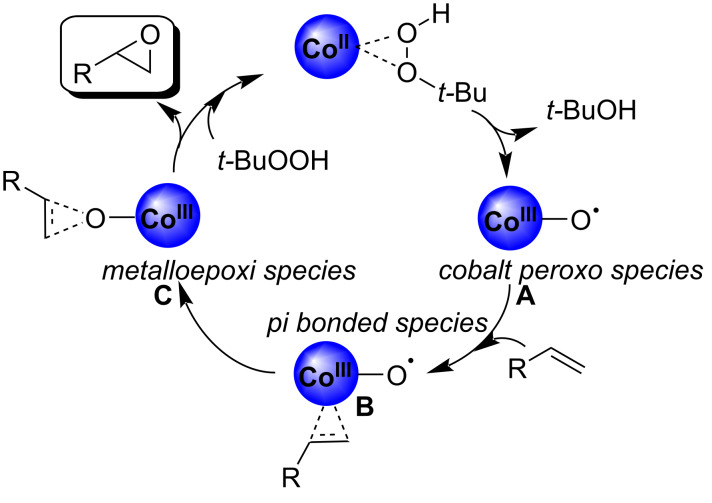
Plausible mechanistic pathway for olefin epoxidation catalyzed by CoNPs/MgO in the presence of *t*-BuOOH.

Based on our results and previous reports by other groups on the same area, we proposed a plausible mechanistic pathway involving a Co(II)/Co(III) couple species, as depicted in Scheme 1. We assume that *tert*-butyl hydroperoxide could react with Co(II) species on the surface of the catalyst leading to the formation of metal-oxy radical **A**. This intermediate could react with the olefin to give π-bonded species of type **B** which could then lead to the formation of the corresponding epoxide product via the metalloepoxi species **C** [55–56]. On the other hand, considering that the reaction is carried out under air atmosphere, and that in the absence of TBHP the epoxidation also takes place to some extent, we assume that a competitive reaction pathway involving the participation of cobalt-superoxo active radicals (Co–O–O•) generated by interaction of the CoNPs with O_2_ [15] could not be disregarded.

## Conclusion

To conclude, we have described a new and efficient heterogeneous CoNPs@MgO catalyst that, in combination with TBHP as oxidant, selectively oxidizes terminal and internal alkenes to the corresponding epoxides in good to excellent yields. The catalyst is readily prepared from commercial starting materials and can be recovered and reused without significant loss of activity. Based on our experimental observations and previously reported studies on cobalt-based alkene epoxidation catalytic systems, a plausible mechanistic pathway involving metal-oxy radical species has been proposed.

## Experimental

All starting materials were of the highest available grade (Aldrich, Fluka, Merck) and were used without further purification. Commercially available cobalt(II) chloride hexahydrate was dehydrated upon heating under vacuum (150 °C, 1.0 mmHg, 45 min) prior to use for the preparation of CoNPs. Column chromatography was performed with Merck silica gel 60 (0.040–0.063 μm, 240–400 mesh). Thin-layer chromatography (TLC) was performed on precoated silica gel plates (Merck 60, F_254_, 0.25 mm).

### Instrumentation and analysis

Nuclear magnetic resonance (NMR) spectra were recorded on a Bruker ARX-300 spectrophotometer using CDCl_3_ as the solvent and tetramethylsilane (TMS) as internal reference. Mass spectra (EI) were obtained at 70 eV on a Agilent HP-7890B GC/MS instrument equipped with a Agilent 5977A selective mass detector. Infrared (FTIR) spectra were obtained on a Nicolet-Nexus spectrophotometer. The purity of volatile compounds and the chromatographic analyses (GC) were determined with a Shimadzu GC-14B instrument equipped with a flame-ionisation detector and a 30 m column (HP-5MS, 0.25 mm, 0.25 μm), using nitrogen as carrier gas.

### Catalyst characterization

The freshly prepared catalyst was characterized by transmission electron microscopy (TEM) in a JEOL 100CX2 instrument, operated at an acceleration voltage of 100 kV. Approximately one hundred metal particles were measured to perform the particle size distribution. The cobalt content in the supported catalyst was determined by inductively coupled plasma atomic emission spectroscopy (ICP-AES), on a Spectro Arcos instrument. X-ray diffraction (XRD) analyses were performed using a Bruker AXS D8 Advance diffractometer, equipped with a Cu-Kα1,2 radiation source. Atomic absorption spectroscopy was carried out on a Perkin Elmer AAnalist200 spectrometer. X-ray photoelectron spectroscopic analyses (XPS) were performed on a PHI 548 spectrometer, using Mg Kα radiation at 250 W and 20 mA. The resolution spectra were taken at 50 eV of pass energy, giving an absolute resolution of ±0.5 eV. The operation base pressure was kept in 10^−10^ Torr range. The adventitious C 1s binding energy was taken as a charge reference and fixed at 284.8 eV.

### Catalysts preparation – general procedure

Analogous as described in [57], a mixture of lithium sand (21 mg, 3.0 mmol) and 4,4'-di-*tert*-butylbiphenyl as electron carrier (DTBB, 26 mg, 0.1 mmol) was placed in a pre-dried Schlenk-type reaction vessel under nitrogen atmosphere. Then anhydrous THF (3 mL) was added and the reaction mixture was stirred at room temperature until it turned dark green (5–10 min), indicating the formation of the corresponding lithium arenide. Anhydrous cobalt(II) chloride was then added (130 mg, 1.0 mmol) and the resulting suspension was stirred until it turned black (15–30 min), indicating the formation of cobalt nanoparticles. Then, it was diluted with THF (10 mL) and 800 mg of the corresponding support (MgO, ZnO, CeO_2_, Celite) were added. The resulting suspension was stirred for 1 h, and then bidistilled water (2 mL) was added for eliminating the excess of lithium. The resulting solid was filtered under vacuum in a Büchner funnel and washed successively with water (10 mL) and acetone (10 mL). Finally, the solid was dried under vacuum (5 Torr).

### Catalytic test – typical procedure for olefin epoxidation

To a vigorously stirred suspension of the CoNPs/MgO catalyst (10 mg) and TBHP (63 μL of an 80 wt % solution in water, 0.5 mmol) in acetonitrile (5 mL), the corresponding alkene (0.5 mmol) was added. The reaction flask was sealed with a screw cap and introduced in a preheated silicon oil bath at a temperature high enough to ensure the reflux of the solvent (82 °C), and stirred at this temperature until total conversion of the starting alkene (TLC, GC). Then, the reaction mixture was centrifuged and the supernatant removed. The solvent was evaporated in vacuo, and the crude product was purified by flash column chromatography (silica gel, hexane/AcOEt). The recovered solid catalyst was washed with acetonitrile (3 × 2 mL) and dried in an oven (150 °C, 1 h) for its reuse.

## Supporting Information

File 1 Detailed experimental procedures, product characterization data, copies of ^1^H and ^13^C NMR spectra of selected epoxides and full characterization of the CuNPs/MgO catalyst.
