# Supplementary material for: A new and efficient methodology for olefin epoxidation catalyzed by supported cobalt nanoparticles
Source: Beilstein J Org Chem. 2021 Feb 22;17:519–26. doi: 10.3762/bjoc.17.46 (PMC7934735; doi:10.3762/bjoc.17.46)
Supplement: File 1 — Detailed experimental procedures, product characterization data, copies of 1H and 13C NMR spectra of selected epoxides and full characterization of the CuNPs/MgO catalyst. [file Beilstein_J_Org_Chem-17-519-s001.pdf]

BEILSTEIN JOURNAL OF ORGANIC CHEMISTRY

Supporting Information

for

A new and efficient methodology for olefin epoxidation catalyzed by supported cobalt nanoparticles

Lucía Rossi-Fernández, Viviana Dorn and Gabriel Radivoy

Beilstein J. Org. Chem. **2021,** *17,* 519–526. doi:10.3762/bjoc.17.46

Detailed experimental procedures, product characterization data, copies of ^{1}H and ^{13}C NMR spectra of selected epoxides and full characterization of the CuNPs/MgO catalyst

License and Terms: This is a supporting information file under the terms of the Creative Commons Attribution License (https://creativecommons.org/licenses/by/4.0). Please note that the reuse, redistribution and reproduction in particular requires that the author(s) and source are credited and that individual graphics may be subject to special legal provisions.
The license is subject to the *Beilstein Journal of Organic Chemistry* terms and conditions: (https://www.beilstein-journals.org/bjoc/terms)

Table of Contents

General experimental details.. SI-2
Instrumentation and analysis.. SI-2
Preparation of the CoNPs/MgO catalyst.. SI-3
Characterization of the CoNPs/MgO catalyst.. SI-3
Figure S1. Representative TEM micrographs of the CoNPs/MgO catalyst.......... SI-3
Figure S2. Selected EDX spectrum of the CoNPs/MgO catalyst.......................... SI-4
Figure S3. XRD pattern of the CoNPs/MgO catalyst.. SI-4
Figure S4. XPS spectrum of the CoNPs/MgO catalyst at the Co 2p level............ SI-5
Representative procedure for the epoxidation of alkenes.................................. SI-5
Product characterization data.. SI-5
NMR spectra of selected epoxides.. SI-8
Figure S5. ^{1}H NMR, ^{13}C NMR and DEPT ^{13}C-NMR of **2a**.................................... SI-8
Figure S6. ^{1}H NMR, ^{13}C NMR and DEPT ^{13}C-NMR of **2b**.................................... SI-10
Figure S7. ^{1}H NMR, ^{13}C NMR and DEPT ^{13}C-NMR of **2d**.................................... SI-11
Figure S8. ^{1}H NMR, ^{13}C NMR and DEPT ^{13}C-NMR of **2h**.................................... SI-13
References.. SI-14

1. General experimental details

Anhydrous tetrahydrofuran was freshly distilled from sodium/benzophenone ketyl. Other solvents were treated before use by standard methods. All starting materials were of the highest available grade (Aldrich, Fluka, Merck) and were used without further purification. Commercially available cobalt(II) chloride hexahydrate was dried in an oven (2 h at 150 °C) and then dried by heating under vacuum until it turned blue, before use. Flash column chromatography was performed with Merck silica gel 60 (0.040–0.063 μm, 240–400 mesh). Reactions were monitored by thin-layer chromatography on silica gel plates (Merck 60, F_{254}) and visualized under UV light and/or using 5% phosphomolybdic acid in ethanol and by CG–MS.

2. Instrumentation and analysis

Nuclear magnetic resonance (NMR) spectra were recorded on a Bruker ARX-300 spectrophotometer using $CDCl_3$ as the solvent and tetramethylsilane (TMS) as internal reference. Mass spectra (EI) were obtained at 70 eV on an Agilent 7890B gas chromatograph coupled with an Agilent 5977A mass selective detector (MSD) (Agilent, Santa Clara, CA), equipped with HP-5MS capillary column (30 m × 0.25 mm inner diameter, 0.25 μm film thickness), using nitrogen as carrier gas. Infrared (FTIR) spectra were obtained on a Nicolet-Nexus spectrophotometer.
The CoNPs/MgO catalyst was characterized by Transmission Electron Microscopy (TEM) on a JEOL 100CX2 instrument, operated at an acceleration voltage of 100 kV. Near one hundred metal particles were measured to perform the particle size distribution. The Energy Dispersive X-ray spectroscopy (EDX) analysis was done on an Electron Microscopy SEM LEO EVO 40 XVP - EDS Oxford X-Max 50 device.
Cobalt content in the supported catalyst was determined by Inductively Coupled Plasma Atomic Emission Spectroscopy (ICP-AES) on a Spectro Arcos instrument. X-ray diffraction (XRD) analyses were performed using a Bruker AXS D8 Advance diffractometer, equipped with a Cu-Kα1,2 radiation source. Atomic Absorption Spectroscopy (AAS) was carried out on a Perkin Elmer AAnalist200 spectrometer. X-ray photoelectron spectroscopic analyses (XPS) were performed on a PHI 548 spectrometer, using Mg Kα radiation at 250 W and 20 mA. The resolution spectra were taken at 50 eV of pass energy, giving an absolute resolution of ±0.5 eV. The operation base pressure was kept in 10^{-10} Torr range. The adventitious C 1s binding energy was taken as a charge reference and fixed at 284.8 eV.

3. Preparation of the CoNPs/MgO catalyst

A mixture of lithium sand (21 mg, 3.0 mmol) and 4,4-di-*tert*-butylbiphenyl (DTBB, 26 mg, 0.1 mmol) in THF (3 mL), was stirred at room temperature under nitrogen atmosphere. When the reaction mixture turned dark green (5–10 min), indicating the formation of the corresponding lithium arenide, anhydrous $CoCl_2$ was added (130 mg, 1.0 mmol). The resulting suspension was stirred until it turned black (15–30 min), indicating the formation of cobalt nanoparticles. Immediately after that, it was diluted with THF (10 mL) and MgO (800 mg) was added. The resulting suspension was stirred for 1 h, and then bidistilled water (2 mL) was added to eliminate the excess of lithium. The resulting solid was filtered under vacuum in a Buchner funnel and washed successively with water (10 mL) and acetone (10 mL). Finally, the solid was dried under vacuum (5 Torr).

4. Characterization of the CoNPs/MgO catalyst

4. a. Transmission electron microscopy (TEM)

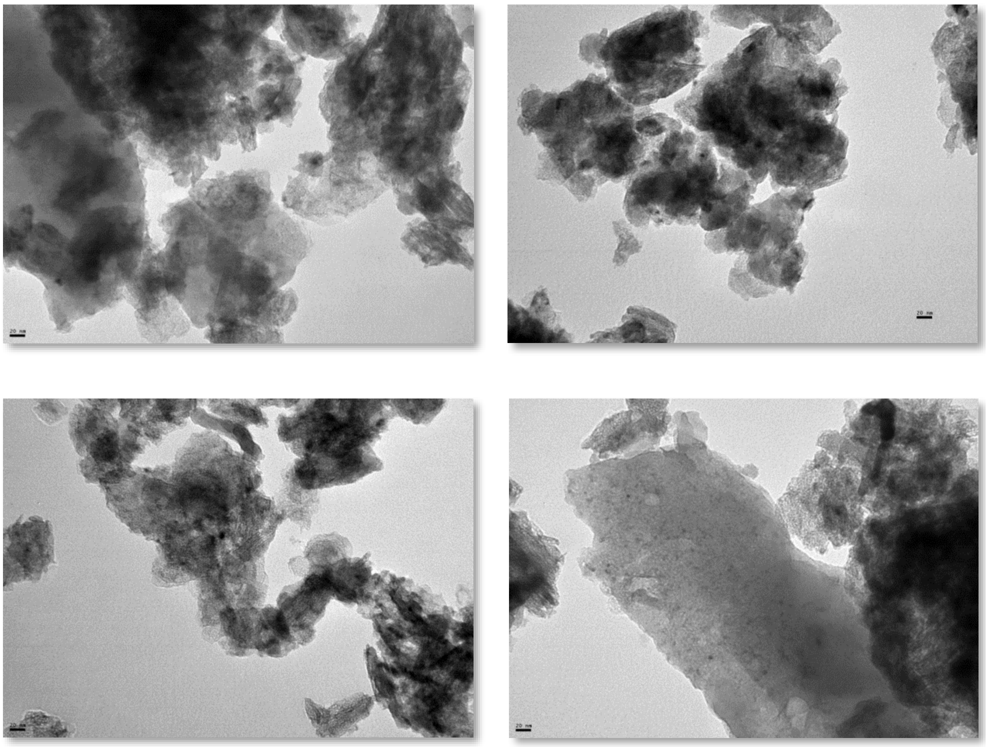

Figure S1. Representative TEM micrographs of the CoNPs/MgO catalyst (scale bar is 20 nm).

4. b. Energy-dispersive X-ray spectroscopy (EDX)

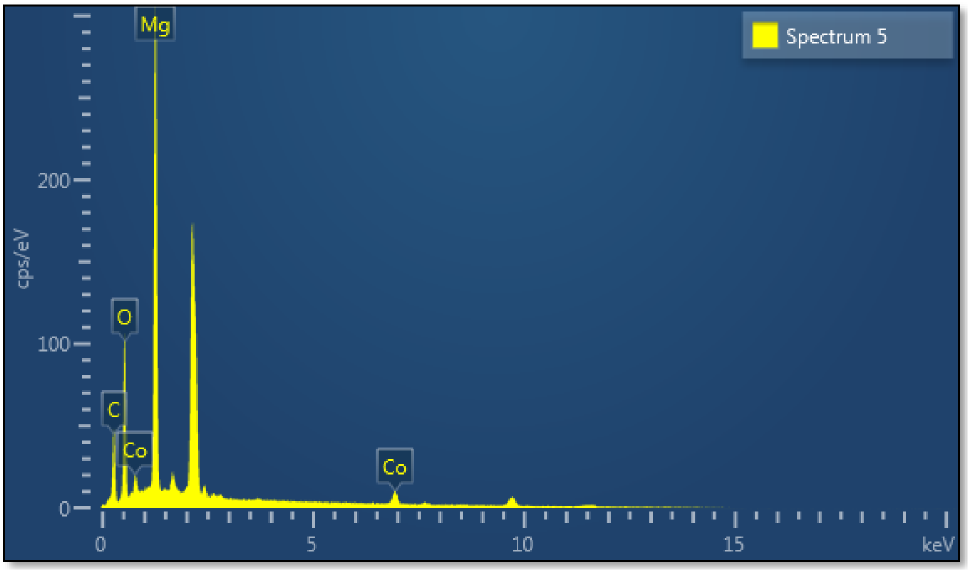

Figure S2. Selected EDX spectrum of the CoNPs/MgO catalyst.

4. c. X-ray diffraction (XRD)

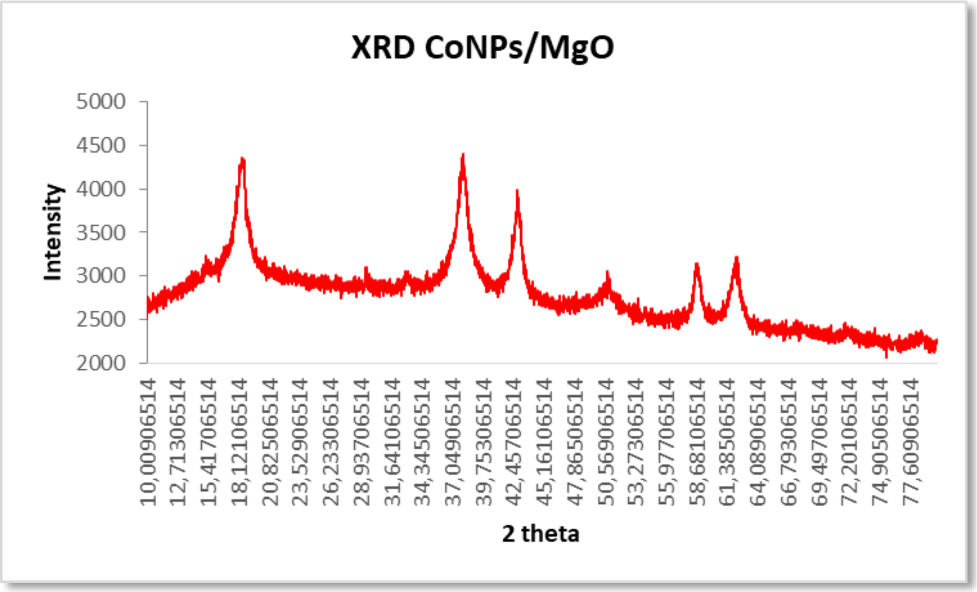

Figure S3. XRD pattern of the CoNPs/MgO catalyst.

4. d. X-ray photoelectron spectroscopy (XPS)

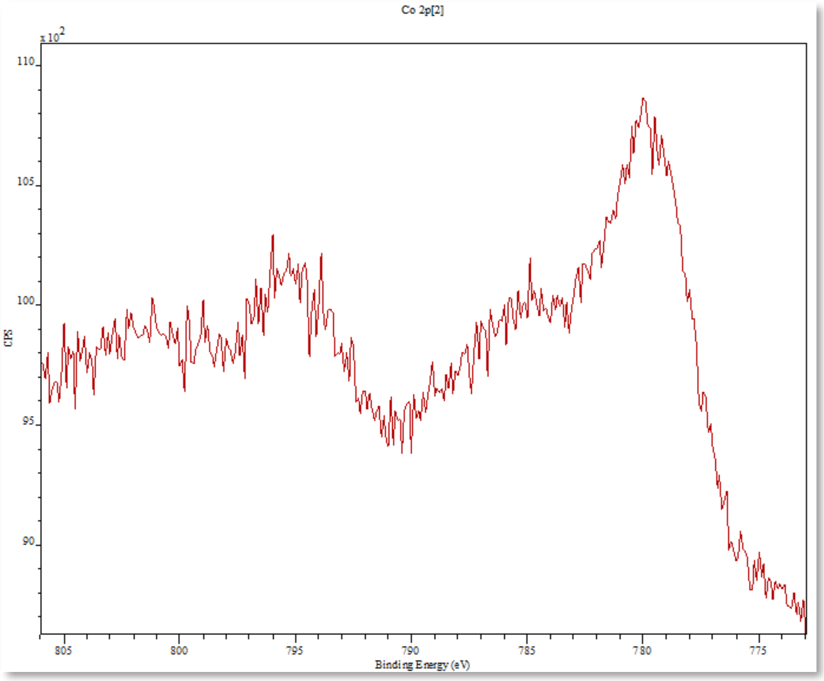

Figure S4. XPS spectrum of the CoNPs/MgO catalyst at the Co 2p level.

5. Representative procedure for the epoxidation of alkenes

The CoNPs/MgO catalyst was activated by heating in an oven at 150 °C for 1 h. To a vigorously stirred suspension of the CoNPs/MgO catalyst (10 mg) in MeCN (1.0 mL) under air the corresponding alkene (0.5 mmol in 1.0 mL MeCN) and TBHP (63 μL of an 80 wt % solution in H_2O, 0.5 mmol in 1.0 mL MeCN) were added and the sealed reaction flask was immersed in an oil bath at the reflux temperature of MeCN (82 °C). The reaction mixture was stirred at this temperature until no further conversion of the starting alkene was observed (TLC, GC). Then, the reaction mixture was centrifuged and the supernatant removed. The solvent was evaporated in vacuo, and the crude product was purified by flash column chromatography (silica gel, hexane/AcOEt) to give the corresponding epoxide. The recovered solid catalyst was washed with acetonitrile (3 × 2 mL) and dried in oven (150 °C, 1 h) for its reuse.

6. Product characterization data

The following known compounds included in Table 2 were characterized by comparison of their chromatographic and spectroscopic data (FTIR, ^{1}H NMR, ^{13}C NMR, and MS) with those described in the literature: styrene oxide (**2a**),[1,2] 1-dodecene oxide (**2b**),[3,4] 2-benzyloxirane (**2c**),[5,6] *trans*-2,3-diphenyloxirane (**2d**),[7,8,9] 2-methyl-3-phenyloxirane (**2e**),[8,10] 1-bromo-2,3-epoxy-3-phenylpropane (**2f**),[11,12] 4-(oxiran-2-yl)phenyl acetate (**2g**),[13,14] 2-methyl-2-phenyl oxirane (**2h**),[7,15] 2,2-

diphenyloxirane (**2i**),[15] 1-phenylcyclohexene oxide (**2j**),[2,7,15] limonene oxide (**2k**),[16,17,18] cyclohex-2-en-1-one (**2l**).[19]

Styrene oxide (2a) [1,2]. Yield: 32 mg (0.45 mmol, 90%); colorless oil. IR (KBr): 3037, 2987, 1606, 1496, 1476, 1451, 1387, 876, 751, 698 cm^{-1}. ^{1}H NMR ($CDCl^3$, 300 MHz): δ = 7.20-7.41 (m, 5H), 3.86 (dd, 1H, J = 4.1, 2.6 Hz), 3.15 (dd, 1H, J = 5.5, 4.1 Hz), 2.80 (dd, 1H, J = 5.5, 2.6 Hz). ^{13}C NMR ($CDCl_3$, 75 MHz): δ = 137.6, 128.5, 128.2, 125.5, 52.4, 51.2. MS (EI, 70 eV): m/z (%) = 120 (20) [M^+], 119 (35), 92 (32), 91 (100), 90 (58), 89 (88), 77 (14), 65 (25), 63 (44), 62 (18), 51 (31).

1-Dodecene oxide (2b) [3,4]. Yield: 38 mg (0.34 mmol, 68%); colorless oil. IR (KBr): 2923, 2854, 1466, 916, 833, 722 cm^{-1}. ^{1}H NMR ($CDCl_3$, 300 MHz): δ = 2.83 (m, 1 H), 2.67 (dd, 1 H, J = 5.2, 4.5 Hz,), 2.39 (dd, 1 H, J = 5.2, 2.6 Hz), 1.54–1.10 (m, 18 H), 0.81 (t, 3 H, J = 6.4 Hz). ^{13}C NMR ($CDCl_3$, 75 MHz): δ = 52.4, 47.1, 32.5, 31.9, 29.6, 29.5, 29.5, 29.4, 29.3, 26.0, 22.7, 14.1. MS (EI, 70 eV): m/z (%) = 184 (0.2) [M^+], 109 (9), 95 (25), 83 (28), 82 (42), 81 (35), 71 (93), 55 (100).

2-Benzyloxirane (2c) [5,6]. Yield: 28 mg (0.35 mmol, 70%); pale yellow oil. IR (KBr): 3028, 2992, 1496, 1455, 1258, 1030, 967, 846, 736 cm^{-1}. ^{1}H NMR ($CDCl_3$, 300 MHz): δ = 7.1–7.2 (m, 5H), 3.1 (dsext, 1H, J = 2.7, 3.9, 5.5 Hz), 2.7–2.9 (m, 3H), 2.5 (dd, 1H, J = 2.7, 5.0 Hz). ^{13}C NMR ($CDCl_3$, 75 MHz): δ = 137.0, 128.8, 128.4, 126.5, 52.3, 46.7, 38.6. MS (EI, 70 eV): m/z (%) = 134 (55) [M^+], 117 (13), 104 (35), 91 (100), 78 (21), 65 (25), 50 (12), 44 (30).

***trans*-2,3-Diphenyloxirane (2d)** [7-9]. Yield: 54 mg (0.46 mmol, 92%); white solid; m.p.: 69-70°C. IR (KBr): 1452, 1278, 860, 837, 745, 690 cm^{-1}. ^{1}H NMR ($CDCl_3$, 300 MHz): δ = 7.41-7.30 (m, 10H), 3.87 (s, 2H). ^{13}C NMR ($CDCl_3$, 75 MHz): δ = 136.8, 128.3, 128.0, 125.2, 62.5. MS (EI, 70 eV): m/z (%) = 196 (25) [M^+], 195 (30), 178 (27), 167 (83), 165 (35), 152 (23), 105 (27), 90 (71), 89 (100), 77 (52), 63 (39), 51 (37).

2-Methyl-3-phenyloxirane (2e) [8,10]. Yield: 36 mg (0.45 mmol, 90%); pale yellow oil. IR (KBr): 3033, 2924, 1691, 1447, 1246, 1067, 687 cm^{-1}. ^{1}H NMR ($CDCl_3$, 300 MHz): δ = 7.20-7.40 (m, 5H), 3.57 (d, 1H, J = 2.1), 3.03 (qd, 1H, J = 5.1, 2.1), 1.45 (d, 3H, J = 5.1). ^{13}C NMR (CDCl3, 75 MHz): δ = 137.9, 128.6, 128.2, 125.7, 59.7, 59.2, 18.1. MS (EI, 70 eV): m/z (%) = 134 (35) [M^+], 133 (44), 117 (8), 105 (52,), 91 (43), 90 (100), 89 (82), 77 (34), 63 (22), 51 (23).

2-(Bromomethyl)-3-phenyloxirane (2f) [11,12]. Yield: 43 mg (0.34 mmol, 68%); colorless solid; m.p.: 46°C. IR (KBr): 2920, 1463, 1380 cm^{-1}. ^{1}H NMR ($CDCl_3$, 300 MHz): δ = 7.25- 7.38 (5H, m), 3.82 (d, 1 H, J = 1.9 Hz), 3.50-3.52 (m, 2 H), 3.31 (dt, 1 H, J = 1.9, 5.8 Hz). ^{13}C NMR ($CDCl_3$, 75 MHz): δ = 136.1, 128.7, 125.8, 60.9, 60.3, 32.0. MS (EI, 70 eV): m/z (%) = 215 (4) [M^+ + 2], 213 (M^+, 4), 171 (19), 169 (19), 105 (18), 91 (100), 89 (25), 77 (20), 63 (20), 51 (18).

4-(Oxiran-2-yl)phenyl acetate (2g) [13,14]. Yield: 34 mg (0.32 mmol, 63%); colorless oil. IR (KBr): 1754, 1507, 1369, 1189, 1165, 1103, 1014, 910, 848, 734 cm^{-1}. ^{1}H NMR ($CDCl_3$, 300 MHz): δ = 7.32-7.25 (m, 2H), 7.11-7.02 (m, 2H), 3.85 (dd, 1H, J = 4.1, 2.5 Hz), 3.13 (dd, 1H, J = 5.5, 4.0 Hz), 2.76 (dd, 1H, J = 5.5, 2.5 Hz), 2.29 (s, 3H). ^{13}C NMR ($CDCl_3$, 75 MHz): δ = 169.5, 150.6, 135.3, 126.7, 121.8, 52.0, 51.3, 21.2. MS (EI, 70 eV): m/z (%) = 178 (5.0) [M$^+$], 136 (35), 135 (20), 121 (20), 119 (8), 107 (100), 78 (18), 77 (24), 51 (16).

2-Methyl-2-phenyloxirane (2h) [7,15]. Yield: 38 mg (0.47 mmol, 94%); colorless oil. IR (KBr): 3012, 2988, 1723, 1495, 1446, 1382, 1343, 1060, 995 cm^{-1}. ^{1}H NMR ($CDCl_3$, 300 MHz): δ = 7.24–7.39 (m, 5H), 2.98 (d, 1H, J = 5.4 Hz,), 2.80 (d, 1H, J = 5.4 Hz), 1.72 (s, 3H). ^{13}C NMR ($CDCl_3$, 75 MHz): δ = 141.2, 128.3, 127.5, 125.3, 57.0, 56.8, 21.8. MS (EI, 70 eV): m/z (%) = 134 (15) [M$^+$], 133 (100), 119 (4), 105 (16), 104 (41), 103 (81), 91 (25), 78 (83), 77 (67), 63 (24), 51 (46).

2,2-Diphenyloxirane (2i) [15]. Yield: 42 mg (0.36 mmol, 72%); white solid; m.p.: 54-56°C. IR (KBr): 2924, 2864, 1742, 1736, 1455, 1375, 1371, 1229 cm^{-1}. ^{1}H NMR ($CDCl_3$, 300 MHz): δ = 7.28–7.46 (m, 10H), 3.39 (s, 2H). ^{13}C NMR ($CDCl_3$, 75 MHz): δ = 57.0, 62.1, 127.6, 128.1, 128.4, 139.7. MS (EI, 70 eV): m/z (%) = 196 (13) [M$^+$], 195 (48), 167 (22), 166 (25), 165 (100), 152 (12), 139 (12), 105 (31), 77 (27), 51 (18).

1-Phenyl-7-oxabicyclo[4.1.0]heptane (2j) [2,7,15]. Yield: 37 mg (0.35 mmol, 70%); colorless oil. IR (KBr): 2937, 1495, 1446, 1359, 1247, 974 cm^{-1}. ^{1}H NMR ($CDCl_3$, 300 MHz): δ = 7.40-7.23 (m, 5H), 3.07 (s, 1H), 2.33-2.08 (m, 2H), 2.02-1.97 (m, 2H), 1.66-1.43 (m, 4H). ^{13}C NMR ($CDCl_3$, 75 MHz): δ = 142.6, 128.3, 127.2, 125.3, 61.9, 60.1, 28.9, 24.7, 20.1, 19.8. MS (EI, 70 eV): m/z (%) = 174 (41) [M$^+$], 173 (55), 159 (13), 145 (22), 129 (47), 117 (51), 115 (75), 105 (79), 103 (38), 91 (80), 77 (100), 65 (16), 51 (43).

1-Methyl-4-(prop-1-en-2-yl)-7-oxabicyclo[4.1.0]heptane (2k) [16-18]. Yield: 24 mg (0.27 mmol, 53%); colorless oil. IR (KBr): 1640, 1250, 950, 810 cm^{-1}. ^{1}H NMR ($CDCl_3$, 300 MHz): δ = 4.75 (s, 2H), 3.02 (t, 1H, J = 5.5 Hz), 1.71 (s, 3H), 1.29 (s, 3H), 1.13–2.27 (m, 7H). ^{13}C NMR ($CDCl_3$, 75 MHz): δ = 148.8, 109.0, 59.2, 57.3, 40.7, 30.7, 28.5, 25.8, 22.1, 20.2. MS (EI, 70 eV): m/z (%) = 152 (1.0) [M$^+$], 137 (25), 123 (10), 109 (35), 93 (45), 79 (51), 67 (100), 55 (31).

Cyclohex-2-en-1-one (2l) [19]. Yield: 25 mg (0.43 mmol, 86%); pale yellow oil. IR (KBr): 3027, 2962, 2924, 2854, 1658, 1597, 1491, 1372, 1255, 1082, 1023, 841, 751 cm^{-1}. ^{1}H NMR ($CDCl_3$, 300 MHz): δ = 6.98 (td, 1H, J = 10.1, 4.2 Hz), 6.00 (td, 1H, J = 10.1, 2.0 Hz), 2.43–2.39 (m, 2H), 2.36–2.32 (m, 2H), 2.04–1.98 (m, 2H). ^{13}C NMR ($CDCl_3$, 75 MHz): δ = 199.8, 150.7, 129.9, 38.1, 25.6, 22.7. MS (EI, 70 eV): m/z (%) = 96 (20) [M$^+$], 68 (100).

7. ^{1}H NMR, ^{13}C NMR and DEPT spectra of selected epoxides

Figure S5. Styrene oxide (**2a**)

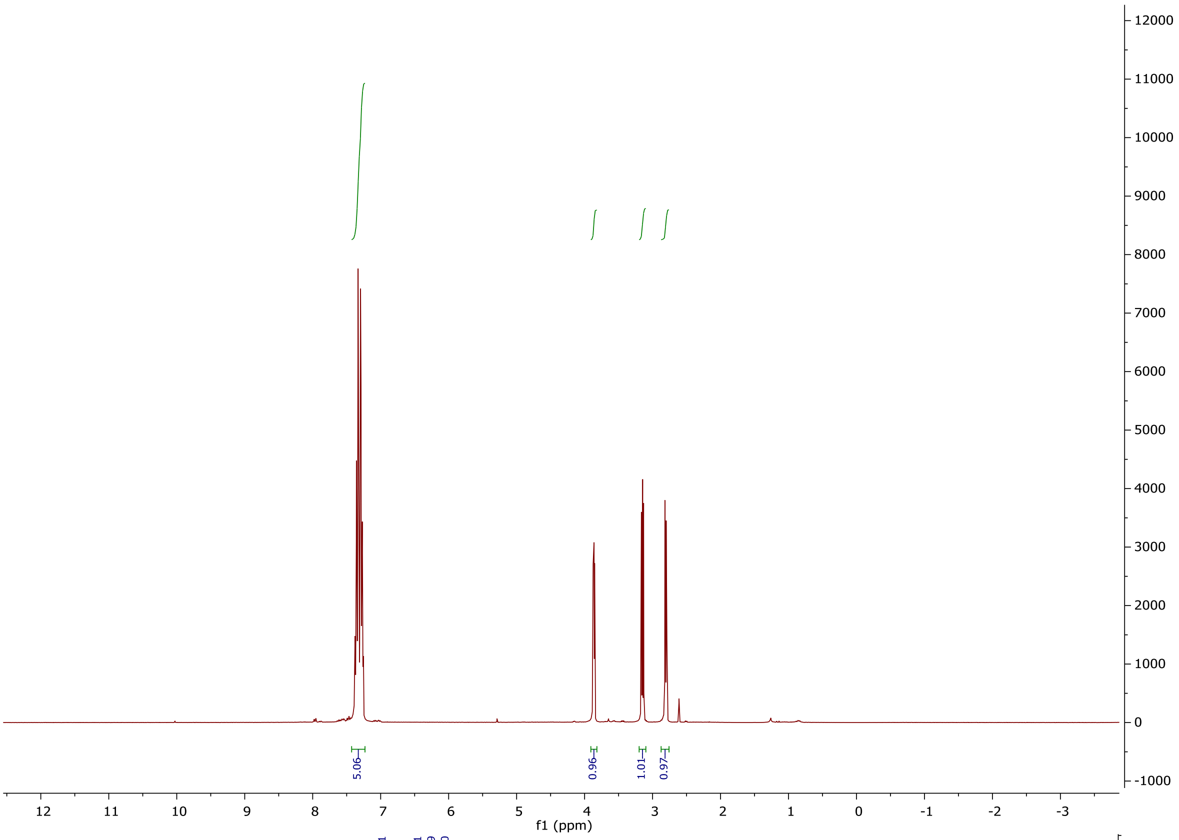

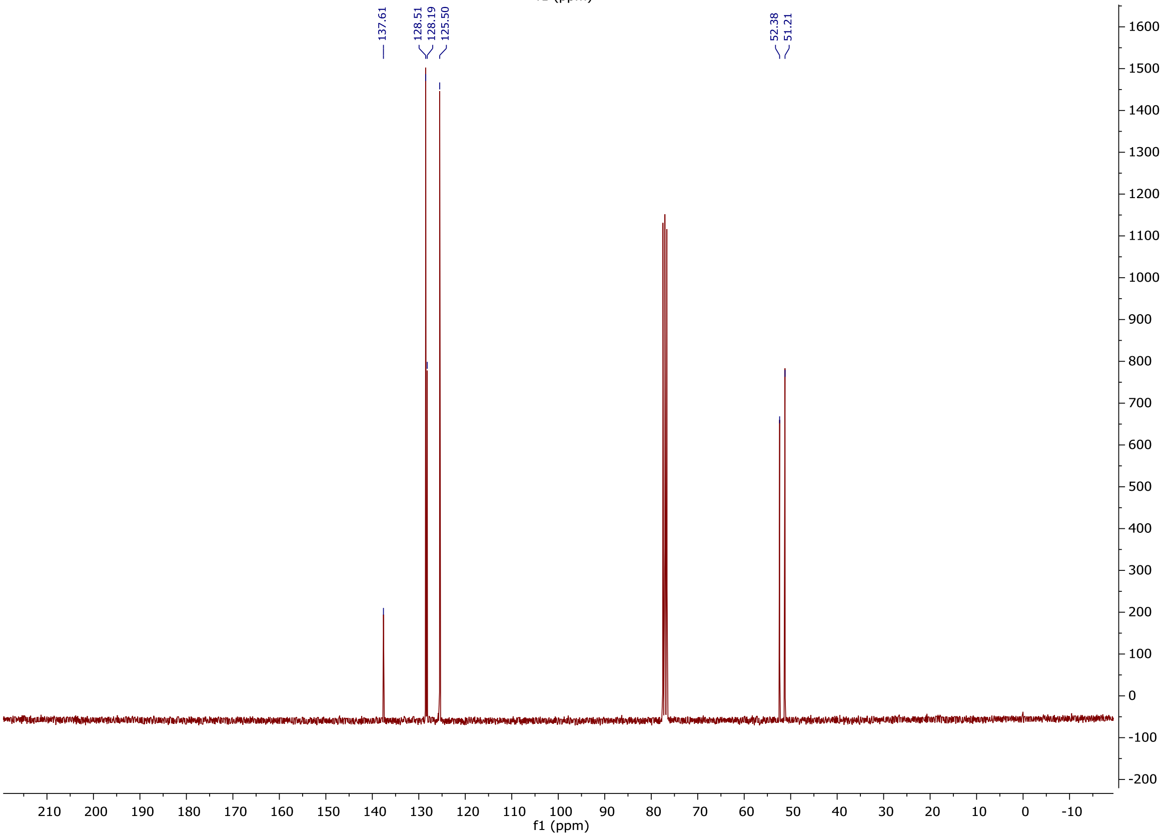

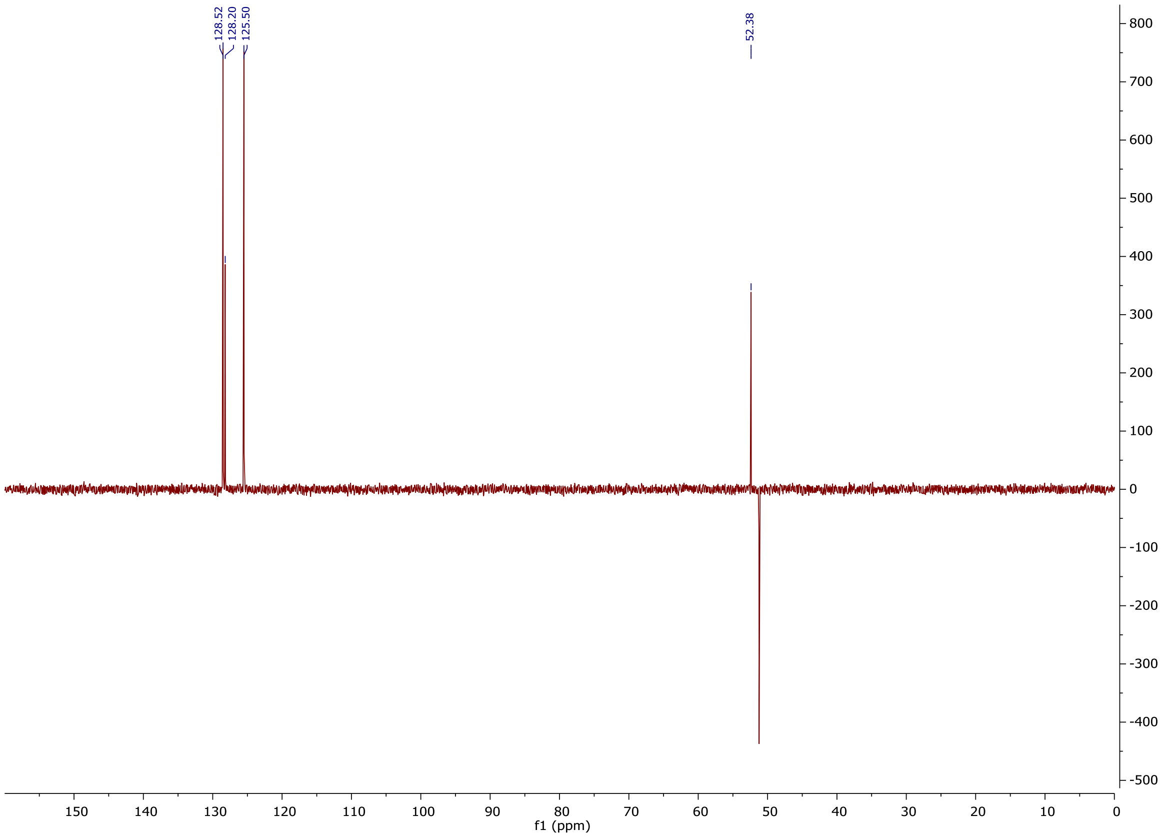
128.52
128.20
125.50
52.38
800
700
600
500
400
300
200
100
0
-100
-200
-300
-400
-500
150
140
130
120
110
100
90
80
70
60
50
40
30
20
10
0
f1 (ppm)

Figure S6. 1-Dodecene oxide (**2b**)

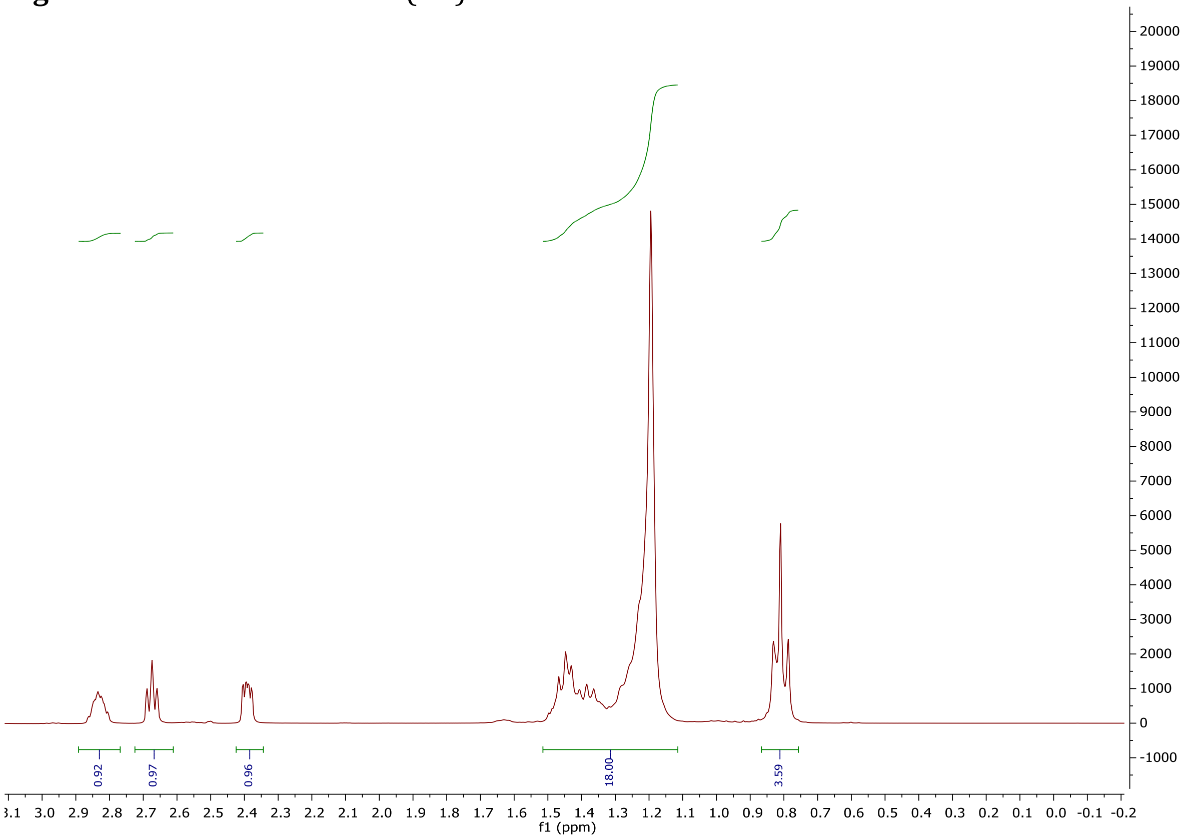

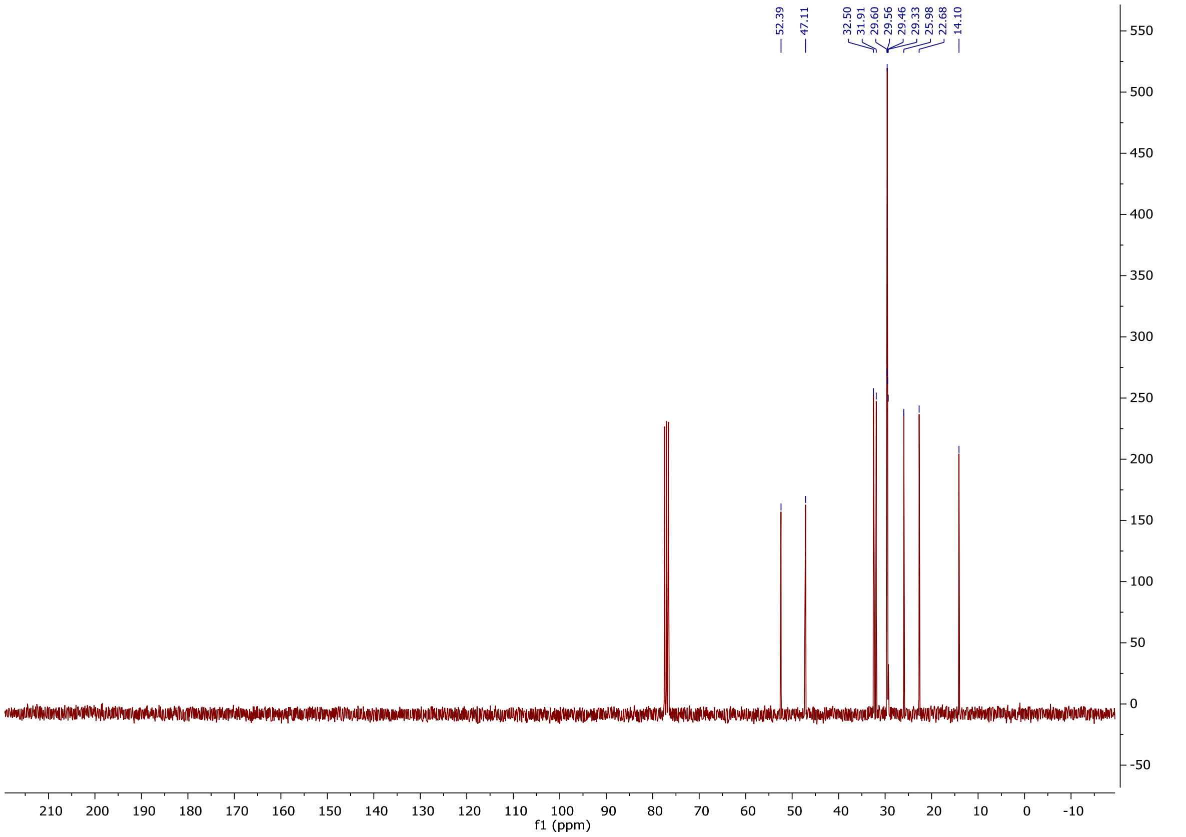

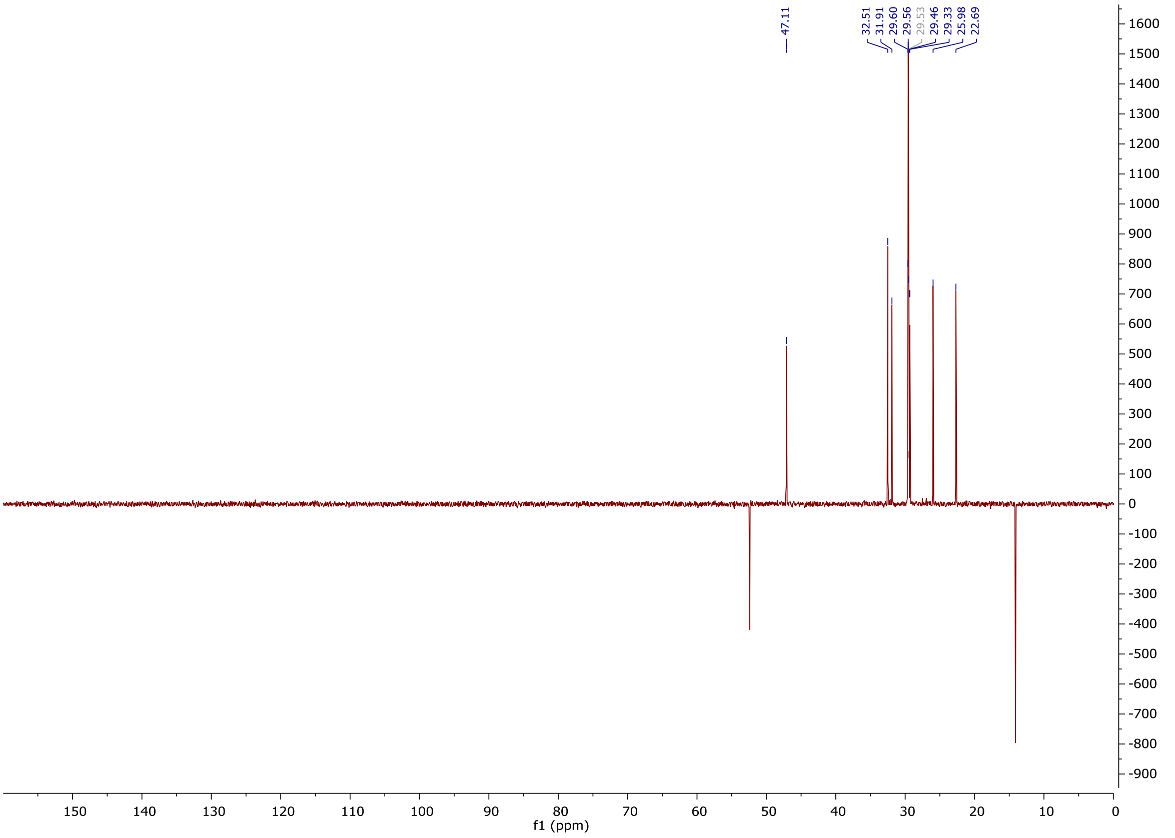

Figure S7. *trans*-2,3-Diphenyloxirane (**2d**)

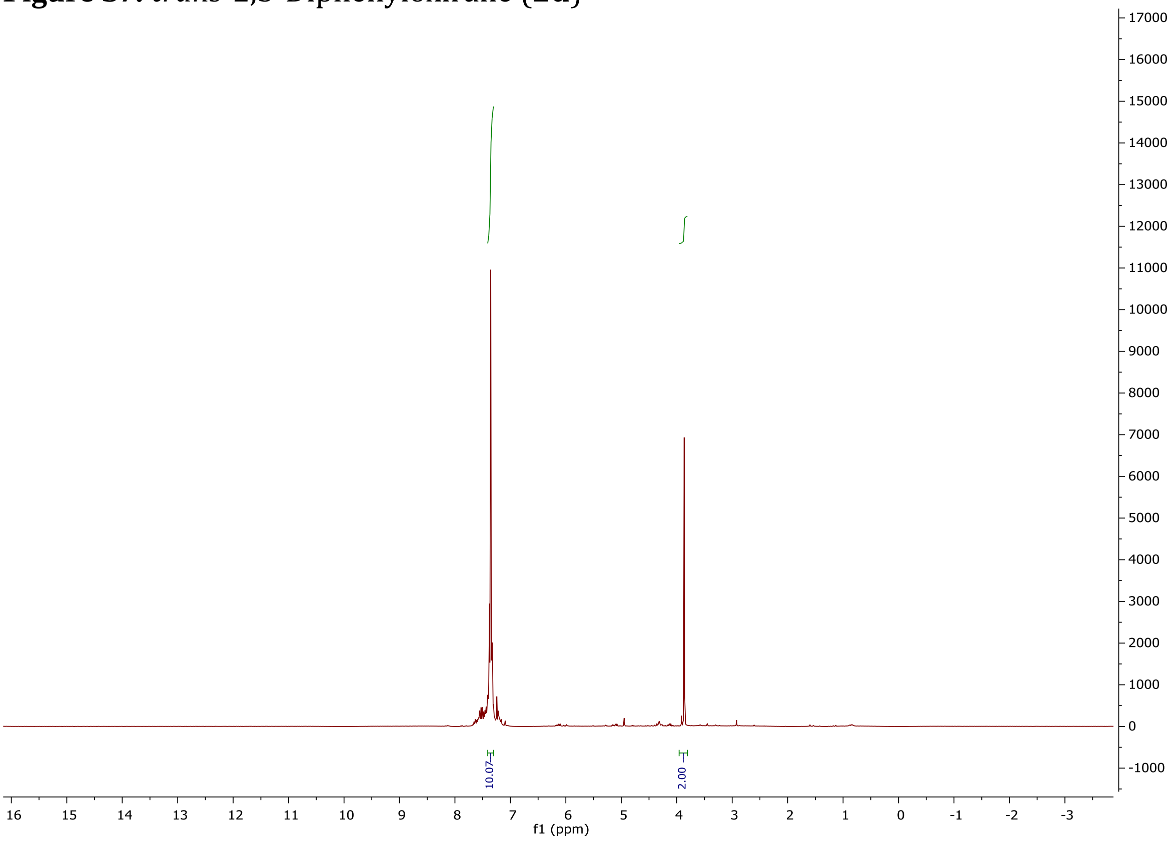

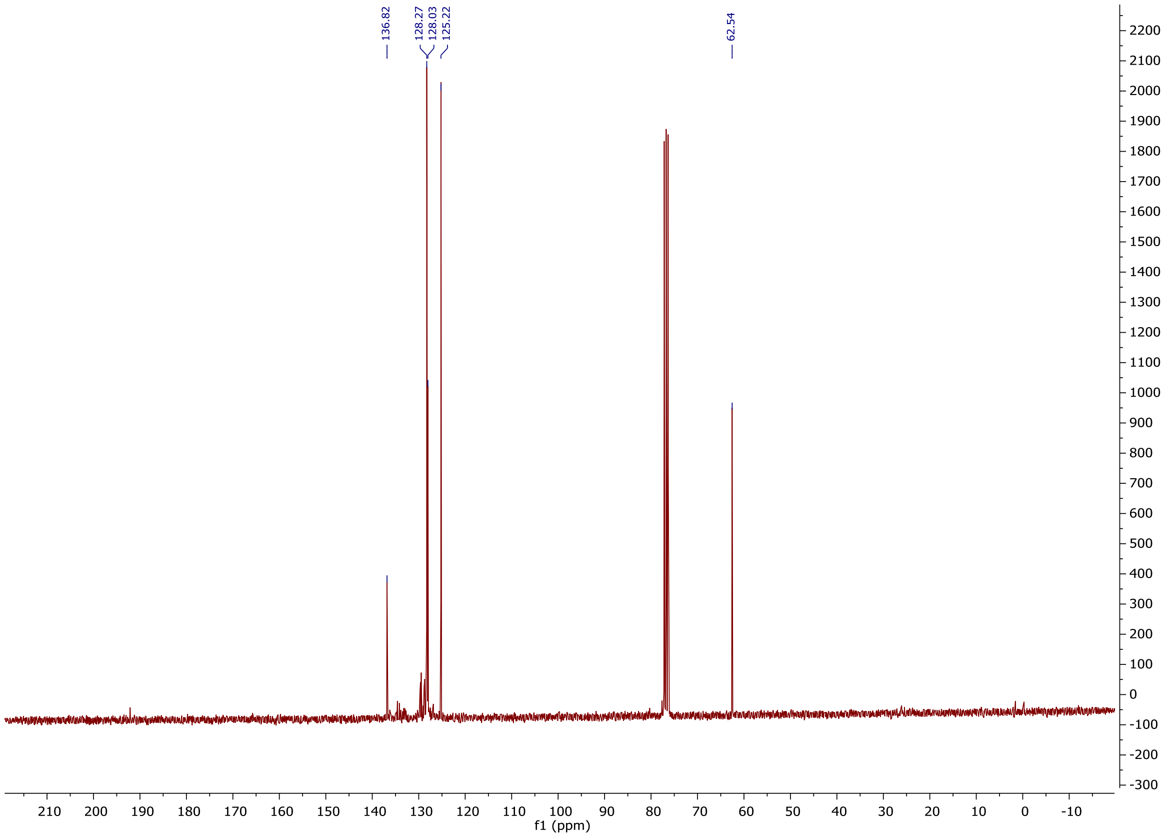
136.82
128.27
128.03
125.22
62.54
f1 (ppm)

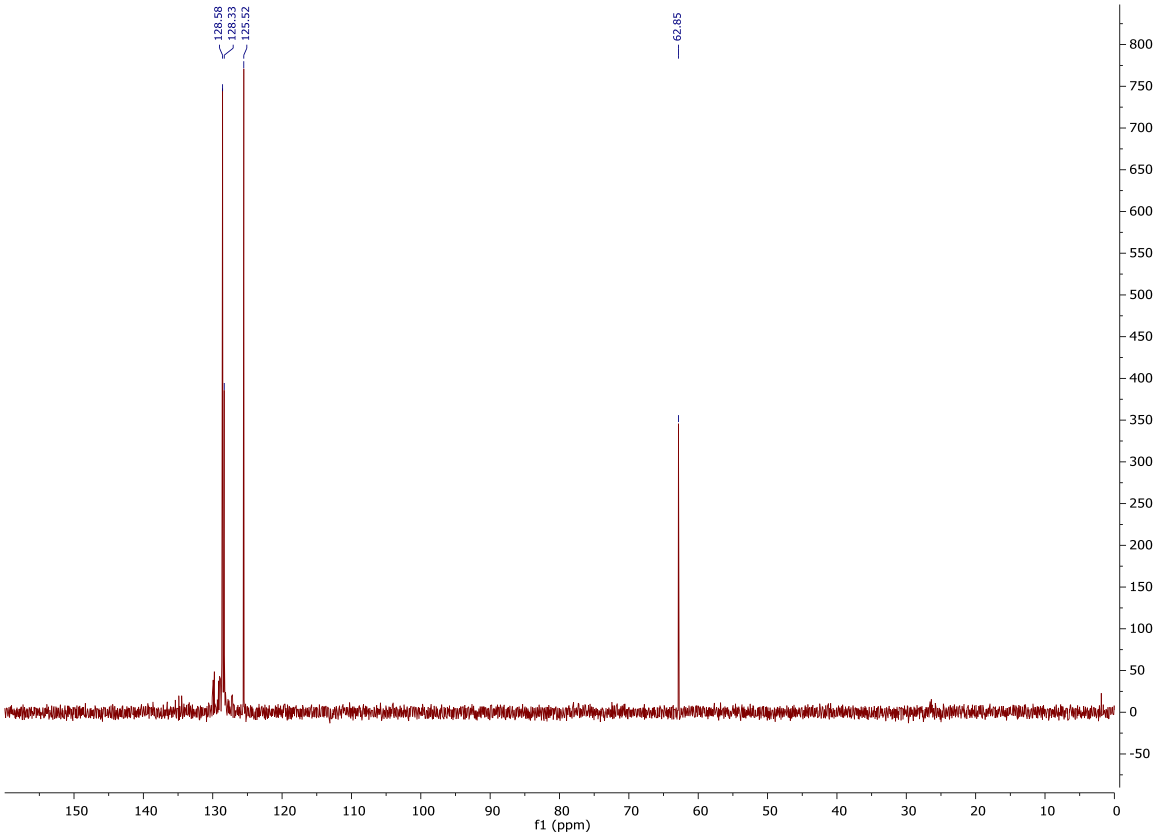
128.58
128.33
125.52
62.85
f1 (ppm)

Figure S8. 2-Methyl-2-phenyl oxirane (**2h**)

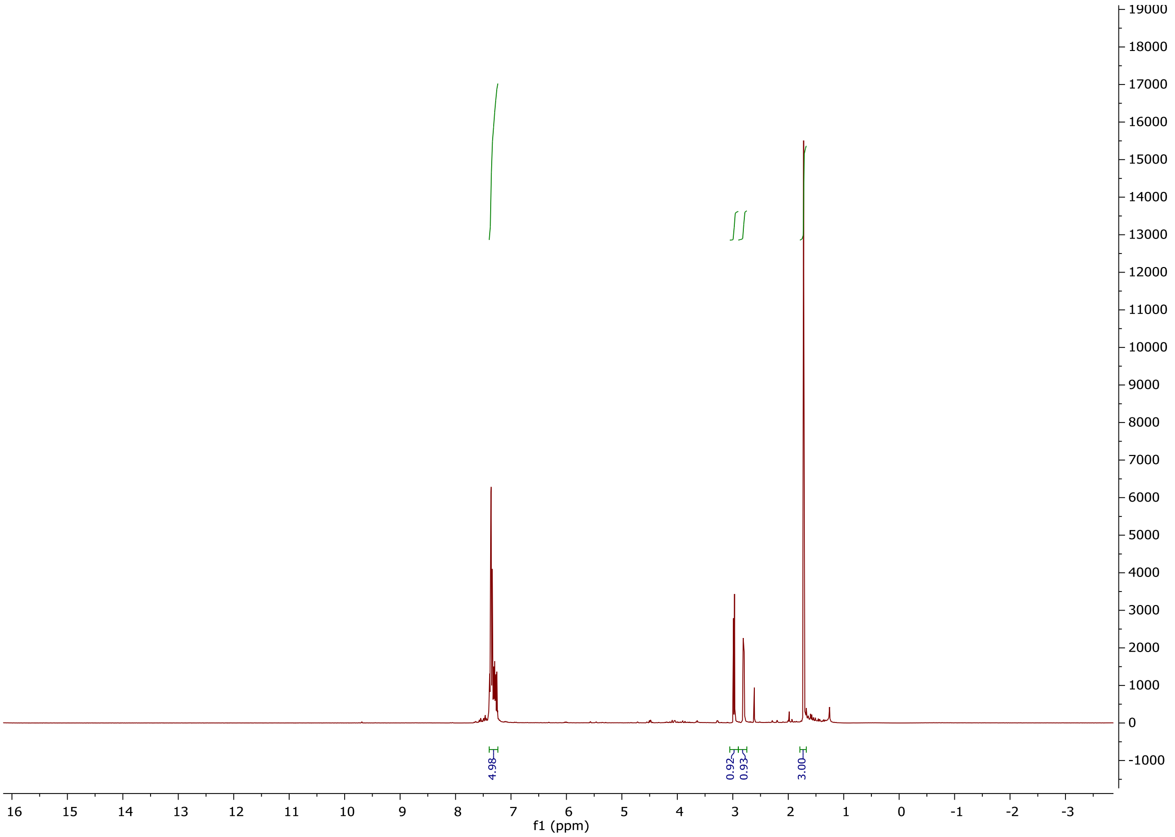

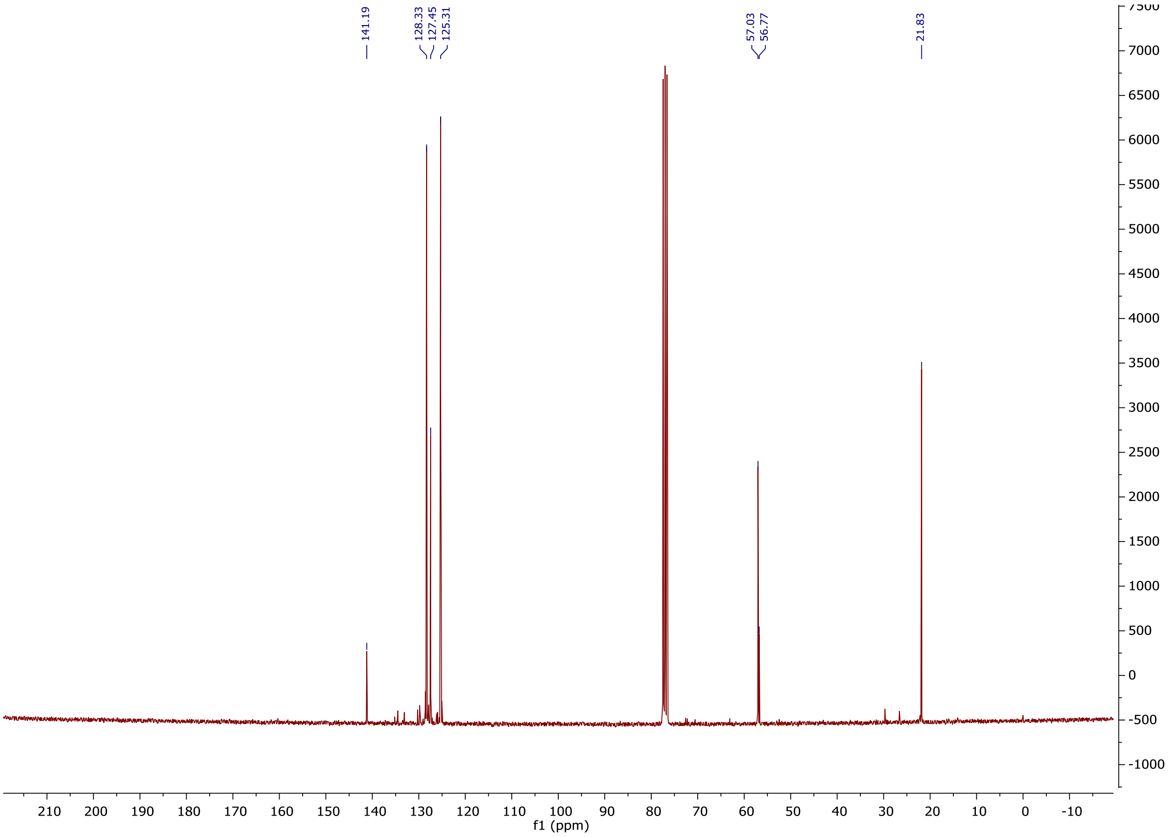

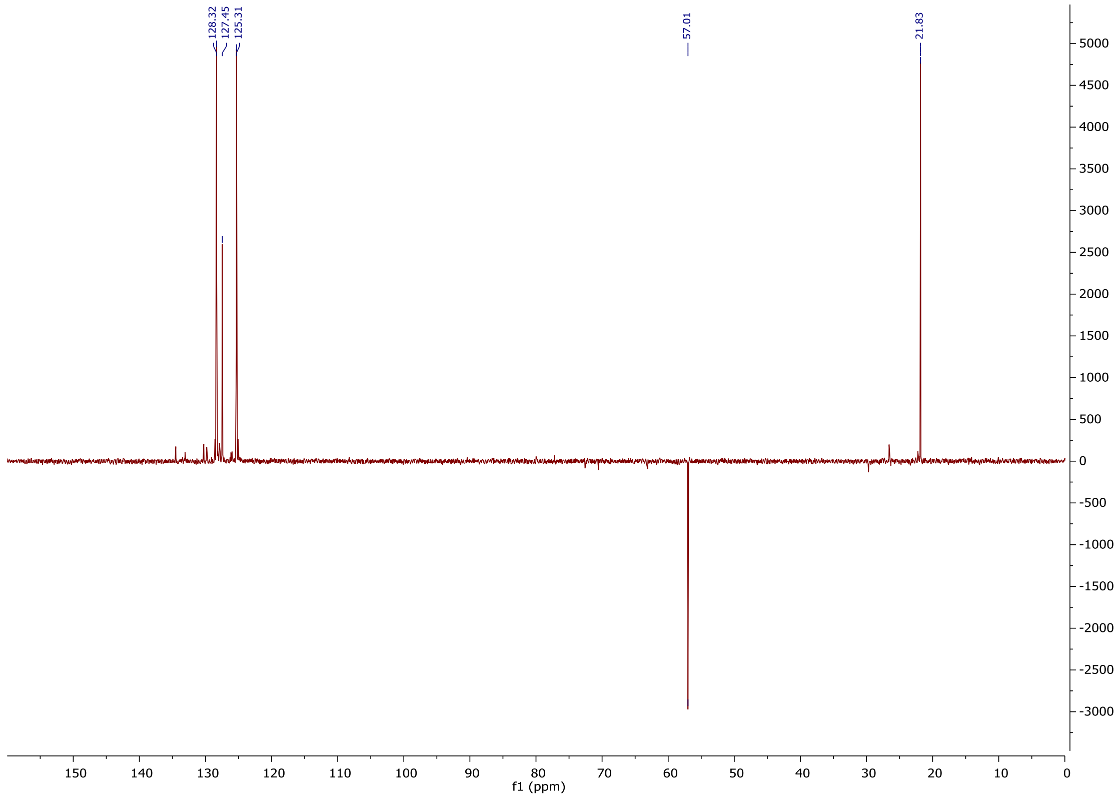

References:

1. A. S. Sharma, H. Kaur, *Appl. Catal. A: Gen.* **2017**, *546*, 136–148.
2. P. C. Bulman Page, B. R. Buckley, L. F. Appleby, P. A. Alsters, *Synthesis* **2005**, *19*, 3405–3411.
3. P. Villo, L. Toom, E. Eriste, L. Vares, *Eur. J. Org. Chem.* **2013**, 6886-6899.
4. C. B. McPake, C. B. Murray, G. Sandford, *Tetrahedron Lett.* **2009**, *50*, 1674–1676.
5. R. Mello, A. Alcalde-Aragones, M. E. González Núñez, G. Asensio, *J. Org. Chem.* **2012**, *77*, 6409–6413.
6. Y. Motoyama, M. Takasaki, S-H. Yoon, I. Mochida, H. Nagashima, *Org. Lett.* **2009**, *11*, 21, 5042-5045.
7. D. Yang, M-K. Wong, Y-C. Yip, X-C. Wang, M-W. Tang, J-H. Zheng, K-K. Cheung, *J. Am. Chem. Soc.*, **1998**, *120*, 5943-5952.
8. X-Q. Li, C. Li, F-B. Song, C. Zhang, *J. Chem. Res.* **2007**, *12*, 722-724.
9. H-T. Chang, K. B. Sharpless, *J. Org. Chem.*, **1996,** *61*, 6456-6457.
10. Z-X. Wang, L. Shu, M. Frohn, Y. Tu, Y. Shi, *Org. Synth.*, **2003**, *80*, 9-17.
11. J. M. Dickinson, J. A. Murphy, C. W. Patterson, N. F. Wooster, *J. Chem. Soc. Perkin Trans. 1* **1990**, 1179-1184.
12. Z. Yu, P. Caldera, F. McPhee, J. J. De Voss, P. R. Jones, A. L. Burlingame, I. D. Kuntz, C. S. Craik, P. R. Ortiz de Montellano, *J. Am. Chem. Soc.* **1996**, *118*, 5846-5856.
13. A. Lahosa, F. Foubelo, M. Yus, *Eur. J. Org. Chem.* **2016**, 4067–4076.
14. M. R. Monaco, D. Fazzi, N. Tsuji, M. Leutzsch, S. Liao, W. Thiel, B. List, *J. Am. Chem. Soc.* **2016**, *138*, 14740–14749.
15. D. J. Vyas, E. Larionov, C. Besnard, L. Guénée, C. Mazet, *J. Am. Chem. Soc.* **2013**, *135*, 6177-6183.
16. C. M. Byrne, S. D. Allen, E. B. Lobkovsky, G. W. Coates, *J. Am. Chem. Soc.* **2004**, *126*, 11404-11405.
17. D. Kaur, R. Manktala, K. K. Chahal, B. R. Chhabra, *Indian J. Chem., Sect B Org. Chem. Incl. Med Chem,* **2010**, *49B-5*, 598-602.
18. Y. Kon, Y. Ono, T. Matsumoto, K. Sato, *Synlett* **2009**, *7*, 1095-1098.
19. M. Zarghani, B. Akhlaghinia, *RSC Adv.* **2016**, *6*, 38592-38601.
